# Sustainable Cement Production: TEA-TIPA as Grinding Aids: Optimizing Ratios for Efficiency and Environmental Impact

**DOI:** 10.3390/polym17192698

**Published:** 2025-10-07

**Authors:** Veysel Kobya, Yahya Kaya, Fatih Eren Akgümüş, Yunus Kaya, Naz Mardani, Ali Mardani

**Affiliations:** 1 Department of Civil Engineering, Faculty of Engineering, Bursa Uludag University, Bursa 16059, Turkey; vkobya@uludag.edu.tr (V.K.); 512126007@ogr.uludag.edu.tr (Y.K.); 502326021@ogr.uludag.edu.tr (F.E.A.); 2 Department of Chemistry, Faculty of Engineering and Natural Sciences, Bursa Technical University, Bursa 16310, Turkey; yunus.kaya@btu.edu.tr; 3 Department of Mathematics Education, Bursa Uludag University, Bursa 16059, Turkey; nazmardani@uludag.edu.tr

**Keywords:** triethanolamine, triisopropanolamine, grinding aid mixtures, grinding efficiency, powder flowability

## Abstract

In line with sustainable construction goals, this study investigates the synergistic use of amine-based grinding aids (GAs), triethanolamine (TEA), and triisopropanolamine (TIPA) to enhance grinding performance and cement properties. GAs were physically blended at varying TEA/TIPA ratios, and their effects on grinding efficiency, CO_2_ emissions, and environmental footprint were assessed based on energy consumption per target Blaine fineness. The interaction of blended GAs with Ca^2+^ ions was modeled to understand adsorption behavior. Cement particle size distribution (PSD), Hausner ratio, Carr index, and angle of repose were analyzed to evaluate powder flowability. Scanning electron microscopy (SEM) was employed to examine microstructural changes. Finally, the Taguchi method statistically analyzed the effective parameters influencing system performance. Results demonstrated that the optimized blend containing 25% TEA and 75% TIPA improved grinding performance, enhanced polymer–ion interactions, refined PSD, and significantly increased powder flowability. Overall, the study underscores the potential of amine-based polymeric GAs in producing environmentally friendly, high-performance cement composites. Using a Taguchi design with the larger-is-better S/N criterion, the optimal formulation was determined to be 25% TEA and 75% TIPA at a dosage of 0.10%. ANOVA results indicated that the TEA content was the most significant factor, while the dosage had no statistically significant effect.

## 1. Introduction

Cement production’s high energy consumption and significant greenhouse gas emissions are among the primary environmental challenges currently facing the industry. While advanced methods such as high-energy-efficient production technologies, carbon capture, and storage (CCS) are employed to mitigate these issues, their widespread adoption is limited due to high implementation costs. Consequently, developing alternative products to cement has emerged as a promising strategy to address these concerns [1,2,3,4].

In parallel, using grinding aids (GAs) in cement manufacturing has become a widely preferred method for enhancing energy efficiency. GAs are dry or liquid chemical additives introduced during cement grinding, providing environmental and economic benefits. Their chemical composition typically includes ethanolamines such as triethanolamine (TEA), monoethanolamine (MEA), and triisopropanolamine (TIPA), as well as glycols like ethylene glycol (EG) and propylene glycol (PG) [5].

TEA is a polar alcohol-amine compound containing both amine and hydroxyl groups. It readily adsorbs onto cement grains and microcracks, reducing surface energy and interfacial tension. Additionally, TEA neutralizes surface charges on cement particles, preventing microcrack closure, particle agglomeration, and coating of mill components [6].

Conversely, TIPA is an alcohol–amine compound with three isopropanol groups and one amine group. Due to its hydroxyl groups, it exhibits strong polarity and acts through a mechanism similar to TEA. However, TIPA has been reported to improve late-age strength more effectively than TEA [7,8,9]. Low surface adsorption capacity and air-entraining effect can negatively affect early-age compressive strength [10,11].

A study by Kong et al. [12] investigated the effects of 0.03%, 0.1%, and 1% TEA dosages on cementitious systems’ 3- and 28-day compressive strengths. The findings showed that TEA positively influenced early-age strength at dosages up to 0.1%, but higher dosages led to reductions in strength. Notably, 28-day strength was adversely affected by TEA across all dosages. He et al. [13] also found that cements ground with 0.02% TEA showed superior early-age compressive strength (3 days) relative to the control sample. Meanwhile, Ma et al. [11] observed that TIPA reduced early-age strength but improved later.

Given these outcomes, the combined use of TEA and TIPA was proposed to mitigate the individual drawbacks of each additive and enhance overall GA performance through synergistic interactions. In this context, Katsioti et al. [14] calculated grinding efficiency via a grindability index (Blaine fineness/mill speed) and found that cement produced using a TEA–TIPA combination exhibited ~20% higher grindability than control cement. Kaya et al. [15] reported that using 0.025% TEA during grinding reduced the cement’s energy demand by 12% compared to the control. Similarly, Gharehgheshlagh et al. [16] showed that cement ground with 0.11% TIPA had a Blaine fineness value ~12% higher than the control at the same grinding duration, indicating improved grindability due to TIPA.

Previous research has shown that TEA enhances early-age strength, while TIPA contributes positively to late-age strength development. However, most studies have focused on the individual effects of TEA and TIPA, and there remains a need to investigate further their impact on grinding efficiency and powder flowability to harness their potential synergistic effects fully. To fill the aforementioned gap, TEA and TIPA were physically blended in five different ratios to evaluate potential synergistic effects arising from their combined individual contributions. Among these, one mixture consisted of 100% TEA and another of 100% TIPA, enabling direct comparison of the blended mixtures with the individual use of each component. These GAs were applied at three different dosages to produce 15 CEM I 42.5R-type cements, alongside a control cement. The cements’ grinding efficiency and particle size distribution (PSD) were evaluated at a target Blaine fineness. CO_2_ emissions and environmental impacts were assessed based on the energy consumed during grinding. The Ca^2+^ adsorption behavior of the GAs was also examined, and the ground products’ powder flowability and morphological characteristics were analyzed. This comprehensive approach allowed for a detailed evaluation of the grinding performance, material properties, and environmental implications of the combined use of TEA and TIPA.

## 2. Materials and Methods

### 2.1. Materials

In this study, the cement samples were produced by grinding a mixture of 96% clinker and 4% gypsum in a laboratory-scale ball mill. Grinding was carried out until the targeted specific surface area of 3900 ± 100 cm^2^/g was achieved. The resulting cements were classified as CEM I 42.5R by the TS EN 197-1 standard. The basic physical and chemical properties of the clinker and gypsum, as provided by the manufacturer, are presented in Table 1.

**Figure 1:**
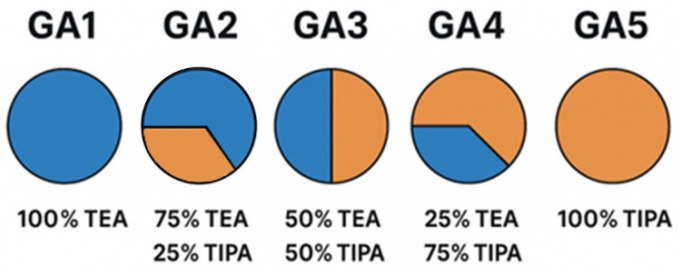
Nomenclature of GAs used and TEA/TIPA mixing ratios.

The physical mixing ratios of TEA and TIPA used as GAs in this study are illustrated in Figure 1.

TEA and triisopropanolamine TIPA are low-molecular-weight alkanolamines. Although not polymers, both are polyfunctional: each contains a tertiary amine and three hydroxyl groups capable of multi-point interactions with ionic species in cementitious pore solutions. Coordination with Ca^2+^ and surface complexation.

The combined amine–alcohol functionality enables multidentate coordination to Ca^2+^, favoring inner-sphere Ca–ligand complexes and adsorption onto clinker/hydration-product surfaces. This multi-site anchoring can generate polymer-like adsorption networks, altering local ion equilibria and modulating early hydration pathways. When combined, TEA and TIPA form cooperative interaction networks: TEA strengthens Ca^2+^ binding while TIPA’s steric profile tunes packing and coverage. The result is an increased effective density of coordination sites, greater stability of Ca^2+^–ligand complexes, and more homogeneous ion distribution at particle interfaces. This coordinated, multivalent binding mimics a key attribute of comb-like PCEs—multi-site anchoring and controlled interparticle spacing—albeit on a low-molecular-weight scaffold. Consequently, TEA–TIPA blends can be treated as polymer-inspired, low-MW analogues that partially replicate dispersive and interfacial-organization effects typical of polymeric admixtures.

### 2.2. Methods

#### 2.2.1. Grinding Process

**Figure 2:**
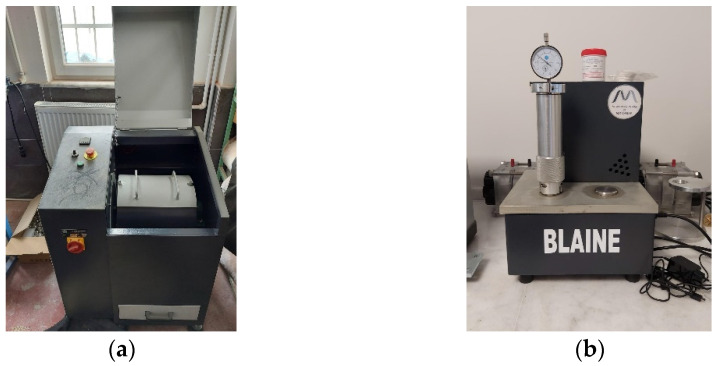
(**a**) Ball mill; (**b**) automatic Blaine device.

The clinker grinding procedure was performed utilizing a Bond-type laboratory ball mill equipped with a 5 kg capacity and powered by a 1.5 kW motor (see Figure 2a). This mill was selected due to its widespread use in the cement industry and its applicability as a reference for designing cement grinding circuits [17]. The ball size distribution recommended by Bond [18] was employed, and 20.13 kg of grinding balls were added to the mill. The corresponding ball filling ratio was calculated as 19.3%. Based on preliminary studies [19], 4 kg of material was used in each grinding test. Besides, the mill was operated at 90% of the critical speed (70 rpm) [19]. The corresponding material filling value for this condition was calculated to be 193% [19].

Grinding was continued for all samples until the desired Blaine of 3900 ± 100 cm^2^/g was achieved. The Blaine of the produced cements was measured using an automatic Blaine device, as shown in Figure 2b. The total grinding cycles needed to attain the specified fineness were systematically recorded. Additionally, the energy consumed by the mill under each grinding condition (ball distribution, mass, and speed) was calculated using Equation (1):

Eg = (220 × Tg × A×1000)/(m × Tg)(1)

Here, Eg represents the grinding energy (kWh/ton), Tg denotes the grinding time (hours), A is the amperage, m is the feed amount (kg), and Tg is the mill factor, which is a fixed value provided by the manufacturer and taken as 4.

#### 2.2.2. Particle Size Distribution

The ground powder samples’ particle size distribution (PSD) was analyzed using laser diffraction with a Hydro 2000S wet dispersion unit integrated into the Malvern Mastersizer 2000. Since powdered materials tend to agglomerate in liquids, achieving homogeneous particle dispersion is essential. Appropriate deagglomeration techniques are required to reduce interparticle bonding forces and ensure effective dispersion. Therefore, ultrasonication was employed, with powders treated ultrasonically in ethanol for 10 min prior to analysis.

#### 2.2.3. Environmental Impact

The environmental impact of grinding aid application during the cement grinding process in Türkiye was assessed in terms of annual electrical energy consumption and associated CO_2_ emissions. For this purpose, national cement production data for Türkiye were used. Based on the obtained values, the environmental impact was quantified according to the energy savings achieved through grinding aids.

#### 2.2.4. Bulk and Tapped Density

Bulk density is a key physical parameter of powdered materials, influenced by processing conditions, storage, chemical composition, particle size, and moisture content. Approximately 5 g of powder was weighed into a 15 mL graduated cylinder to determine bulk density, and its volume was recorded. Bulk density (g/mL) was then calculated by dividing the mass by the measured volume.

Tapped (distributed) density was measured on the same sample. The powder in the graduated cylinder was compacted by vibrating it 150 times at a constant speed. After vibration, the new volume was measured, and tapped density (g/mL) was calculated using the same mass-to-volume ratio.

#### 2.2.5. Hausner Ratio and Carr Index

The Hausner ratio and Carr index are widely used to evaluate the flowability of powders. Bulk and tapped densities are nearly equal in free-flowing powders, resulting in a low Carr index. However, greater differences between bulk and tapped densities are observed in powders with poor flowability and stronger interparticle interactions, yielding a higher Carr index. These parameters were calculated using Equations (2) and (3), based on the measured bulk and tapped density values.

(2) $$CI=\frac{DY-YY}{DY}$$

(3) $$HR=\frac{DY}{YY}$$

Here, DY represents the distributed density and YY represents the bulk density.

#### 2.2.6. Angle of Repose

Angle of repose measurements were conducted according to the specified procedures. A fixed mass of powder was allowed to flow from a hopper with a 60° cone angle and a 5 mm orifice onto a flat surface, forming a conical pile with a constant base diameter (fixed cone method). The vertical distance between the hopper’s outlet and the base surface was maintained at 80 mm throughout the test.

#### 2.2.7. Fineness Modulus

The Fineness Modulus (FM) is a parameter used to represent the particle size distribution and provides insight into the average fineness of cement. To assess the relationship between the structural characteristics of the cement powder and its fineness, FM was calculated using Equation (4):

(4) $$FM=\sum_{i=1}^{300}cumulative\;passing\;rate/100$$

Here, i represents the selected particle size.

#### 2.2.8. Theoretical Calculations

In this study, the molecular structures of TEA (triethanolamine), TIPA (triisopropanolamine), the TEA-TIPA mixture, and their interactions with Ca^2+^ ions were modeled using the GaussView 5.0 software. Geometry optimizations and frequency calculations for all molecular systems were carried out using the Gaussian 09 software package. The absence of imaginary frequencies in the vibrational analysis confirms that the optimized structures correspond to true minima on the potential energy surface. All optimizations were performed using the semiempirical PM6 basis set, which provides a good balance between computational efficiency and accuracy, particularly for systems containing large organic molecules and metal ions.

In the adsorption calculations, a single Ca^2+^ ion was positioned approximately 5 Å away from each organic molecule, and the total charge of the system was set to +2. All calculations were conducted under gas-phase conditions at room temperature (298 K). During the geometry optimization, all atoms in the system were fully relaxed without any geometric constraints, allowing the system to reach its most stable conformation on the potential energy surface.

#### 2.2.9. Experimental Design Using the Taguchi Method

In this study, the Taguchi method and analysis of variance (ANOVA) were employed to identify the optimal combination of grinding parameters. The Taguchi approach utilizes different signal-to-noise (S/N) ratio calculation strategies—namely, nominal-is-best, larger-is-better, and smaller-is-better—based on the nature of the response characteristic [19]. Here, the “larger-is-better” criterion, presented in Equation (5) and recommended by Mandal et al. [20], was adopted as the objective function.

(5) $$\frac{S}{N}=-10log(\frac{1}{n}\;\sum_{i=1}^{n}{1/yi}^{2})$$

where yi presents the data observed in the i-th experiment, and n denotes the number of observations.

The control factors were defined as the type and dosage of the grinding aid (GA). The levels corresponding to these factors are presented in Table 2. To identify the optimal grinding performance and evaluate the influence of each factor, the L15(5^1^ 3^1^) orthogonal array was selected as the most suitable experimental design.

## 3. Results and Discussion

### 3.1. Theoretical Calculations

**Figure 3:**
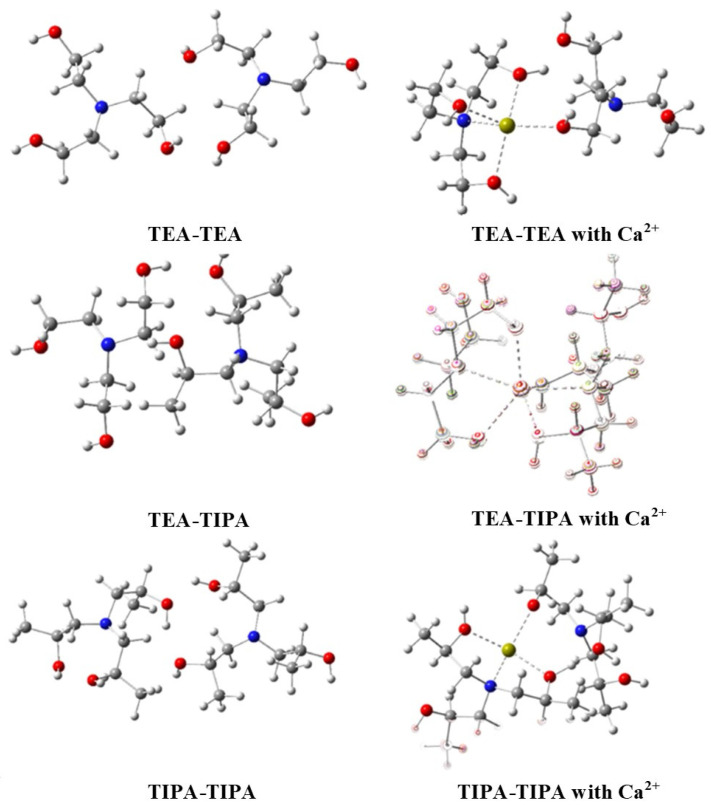
Optimized structure of TEA, TIPA, TEA-TIPA molecules, and molecules adsorbed with Ca^2+^ ion. *(Red: oxygen, Yellow: calcium, Blue: nitrogen, Grey: carbon, White: hydrogen)*.

The optimized structures of TEA-TEA, TEA-TIPA, and TIPA-TIPA molecules and their Ca^2+^-adsorbed forms were calculated using the semiempirical PM6 method and are presented in Figure 3. To elucidate the synergistic effects of the molecular mixtures, dimeric structures of TEA-TEA, TEA-TIPA, and TIPA-TIPA were analyzed. The interactions between the optimized dimers and Ca^2+^ ions were also investigated. The corresponding optimized and interaction energies are summarized in Table 3. Figure 3 shows 5-, 6-, and 4-fold coordination of Ca^2+^ ions were observed in the optimized adsorption structures of TEA-TEA, TEA-TIPA, and TIPA-TIPA, respectively. Notably, the six-fold coordination observed in the TEA-TIPA structure, involving both molecules in the adsorption complex, led to enhanced adsorption strength. Accordingly, the TEA-TIPA model exhibited the highest adsorption energy, which was calculated as 0.402345. These findings support the experimental results and confirm the synergistic interaction effect within the TEA-TIPA mixture.

### 3.2. Grinding Efficiency, Particle Size Distribution, and Environmental Impact

Under this heading, the grinding efficiencies achieved using different proportions of TEA and TIPA as grinding aids (GAs) are discussed, along with the resulting benefits regarding grinding performance and the corresponding particle size distribution of the cement.

#### 3.2.1. Grinding Efficiency

**Figure 4:**
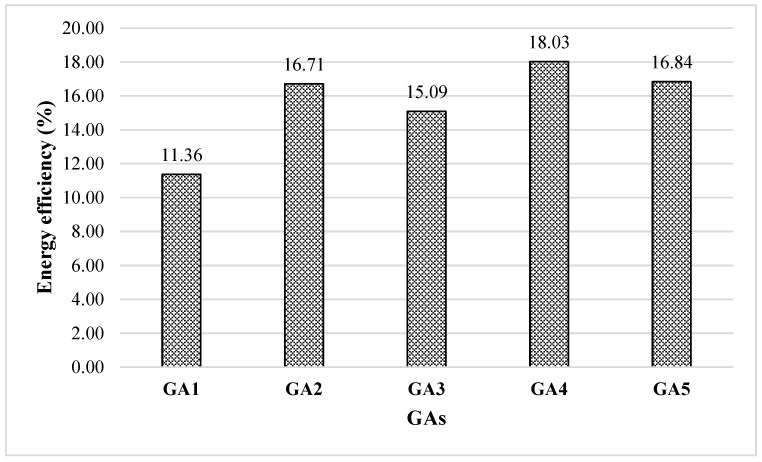
Average energy efficiency of GAs.

The grinding efficiencies of the GAs used in the study at different dosages are given in Table 4, and the average energy efficiencies are given in Figure 4.

Table 4 demonstrates that the type and dosage of grinding aids (GAs) significantly influence the grinding time, energy consumption required to achieve the target Blaine fineness, and the resulting relative energy efficiency. As illustrated in Figure 4, GAs markedly enhance energy efficiency across all additive types and dosages. Among them, the TEA-only formulation (GA1) yielded the lowest relative energy efficiency at 11.36%, whereas efficiency progressively increased with higher TIPA content. This improvement is attributed to TIPA’s more effective dispersion capability, which reduces interparticle cohesion and lowers the energy demand during grinding [6].

The highest relative energy efficiency, 18%, was obtained using GA4, a blend comprising 25% TEA and 75% TIPA. This outcome suggests that TEA and TIPA operate via distinct mechanisms within the cement matrix and exhibit a synergistic effect when combined in optimal ratios. TEA is known to mitigate particle agglomeration by decreasing electrostatic interactions, facilitating more effective particle breakage [5,19,21]. However, this effect tends to plateau with increasing dosage and becomes limited when TEA is used in isolation. Previous studies indicate that TEA’s action is primarily based on its interaction with Ca^2+^ ions at the particle surface, thus restricting its effect to surface modification [22].

**Figure 5:**
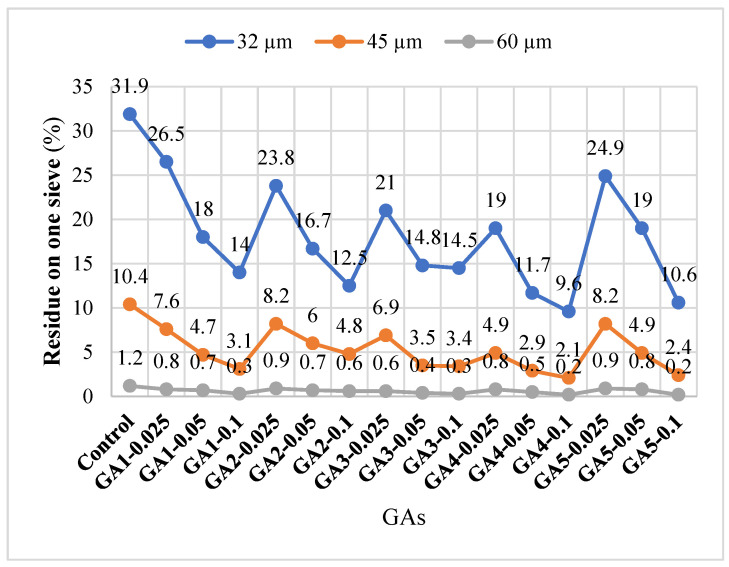
Particle size distributions of cements produced with GA.

On the other hand, TIPA enhances specific surface area not only through surface interactions but also by promoting a more stable dispersion of fine particles. This is attributed to its strong complexing ability, which prevents the re-agglomeration of particles formed during grinding [22,23]. Consequently, TIPA appears more effective than TEA at higher dosages. As illustrated in Figure 5, TEA–TIPA combinations demonstrated superior grinding performance by synergistically integrating the individual benefits of both additives. Specifically, GA4 delivered the most effective performance by reducing interparticle cohesion through the action of TEA and suppressing the re-agglomeration of fine particles through the effect of TIPA (Table 4, Figure 5). Consequently, GA4 enhanced grinding efficiency for the target Blaine fineness by 16.6–18.3% compared to the control (Table 4). This improvement is attributed to the combined mechanisms: the electrostatic interactions of TEA and the steric hindrance imparted by the methyl groups of TIPA.

Similar observations were reported by Mao et al. [22], who found that TEA–TIPA mixtures, when used at specific ratios, improved grinding energy efficiency by 10–20% compared to single-component systems. In the present study, the maximum observed improvement of 18% aligns well with these findings and highlights the importance of admixture optimization.

Although GA5 (100% TIPA) exhibited high grinding efficiency, its performance (16.8%) was slightly lower than that of GA4. This difference may be attributed to TIPA saturation at elevated dosages and an additive–matrix interaction limitation. These results suggest that the effectiveness of grinding aids depends not only on their chemical structure but also on the balance of their physicochemical interactions with cement particles [24].

Kobya et al. [21] evaluated the grinding efficiencies of TIPA, DEIPA, DEG, and EG-type GAs at comparable dosages, targeting a Blaine fineness of 4100 ± 100 cm^2^/g under similar conditions. Reported average efficiencies relative to the control were 18.6%, 9.7%, 12.6%, and 8.3% for TIPA, DEIPA, DEG, and EG, respectively. In the present study, GA4 (75% TIPA + 25% TEA) exhibited higher performance (Figure 4) than all GAs reported by Kobya et al. [21], except for TIPA alone. This finding supports the previously discussed synergistic advantages of combining TEA and TIPA.

In conclusion, the optimized use of TEA and TIPA combinations significantly improves grinding efficiency, offering potential reductions in energy consumption and CO_2_ emissions within cement production.

#### 3.2.2. Environmental Impact

Based on the collected data, the impact of grinding aid application on annual electrical energy consumption and related CO_2_ emissions in the cement grinding process in Türkiye was evaluated. A summary of the estimated results is presented in Table 5.

As shown in Table 5, approximately 84 kilotons of cement were produced in Türkiye in 2024 [25]. Using GAs during this production could result in energy savings of approximately 306 to 598 GWh. Based on national emission factors, where 0.478 kg of CO_2_ is emitted per kWh of electricity consumed, applying GAs could prevent an estimated 146 to 286 kilotons of CO_2_ emissions [26].

Considering that a single tree can absorb about 12 kg (0.012 tons) of CO_2_ annually through photosynthesis [27], this reduction corresponds to the annual sequestration capacity of approximately 12 to 23 million trees. For comparison, clinker grinding without using GAs emits around 1500 kilotons of CO_2_ per year, equating to the CO_2_ absorption capacity of roughly 125 million trees.

Moreover, based on data from the literature [28], the average annual electricity consumption of a four-person household in Türkiye is approximately 5000 kWh. Thus, the energy savings achieved through GA usage could meet the yearly electricity needs of 61,000 to 120,000 households.

Lower-carbon production can be achieved by partially replacing Portland cement with alternative binders and/or recycled constituents [1,2,3,8]. The CO_2_ benefit from clinker reduction typically exceeds the savings attainable from finish-grinding alone because it directly avoids process (calcination) emissions. Complementary to this, our results indicate that grinding in the presence of GA not only lowers specific energy demand but also refines the particle size distribution and improves powder flow. These effects can enable higher levels of pozzolanic/SCM substitution at equivalent performance, thereby amplifying the environmental gains of clinker reduction. In this sense, GA-processed cements provide an enabling platform for more aggressive pozzolanic substitution regimes and, by extension, more sustainable concrete production.

#### 3.2.3. Particle Size Distribution

Particle size distribution during cement grinding is a crucial factor that directly influences both cement hydration kinetics and the performance of GAs. This study examined the impact of TEA and TIPA, used in various ratios as GAs, on particle size distribution. Particular attention was given to the percentages of particles retained on 32 µm, 45 µm, and 60 µm sieves, as illustrated in Figure 5.

GAs significantly increased the fraction of particles below 32 µm, enhancing grinding efficiency compared to the control sample without additives.

Moreover, analysis of the particle fractions passing below 45 µm and 60 µm reveals that GAs effectively reduce coarse particles. The pass rate below 45 µm, which was 31.9% in the control sample, decreased markedly in all GA-containing mixtures. The lowest value, 2.1%, was observed in the GA4-0.1 sample (Figure 5). This behavior observed in GA4, which consists of 75% TIPA and 25% TEA, aligns with the findings of Mao et al. [22]. They reported that TIPA effectively dissolves agglomerates and minimizes the film-forming effect on cement surfaces. Additionally, Altun et al. [29] demonstrated that TEA reduces the likelihood of agglomeration by enhancing electrostatic repulsion between particles, thereby improving grinding efficiency.

A notable observation is that increasing the GA dosage consistently results in finer particle distributions. This suggests that higher dosages enhance the impact-grinding mechanism, where the lubricant effect of the additive dominates, thereby improving grinding medium efficiency [30].

Additionally, sub-60 µm pass values remaining below 1% in nearly all GA systems indicate effective suppression of coarse fractions. This demonstrates that the additives contribute to a more homogeneous particle size distribution, which increases fineness and potentially boosts cement reactivity by narrowing the particle size distribution curve [31].

Consequently, TEA and TIPA additives, individually and in combination, positively influence particle size distribution. However, determining the optimum additive ratio and dosage is crucial. The appropriate balance enhances energy efficiency during grinding, producing a more stable and reactive binder with improved performance.

### 3.3. Powder Flow Properties of Cement

Parameters, including bulk density, tapped density, Carr index, Hausner ratio, and angle of repose, were measured to assess the flow properties of the ground cements. The flowability values corresponding to these parameters, based on classification scales reported in the literature [32,33], are summarized in Table 6.

#### 3.3.1. Hausner Ratio and Carr Index

Flowability assessment was conducted following the classification scale proposed by Carr [32]. Flowability refers to the ability of a powder to flow under specified conditions, considering factors such as applied pressure and ambient humidity. Table 7 presents the bulk densities of the cements measured at a constant mass without vibration, the tapped densities obtained after vibration, and the corresponding Carr index and Hausner ratio values.

The control cement exhibited the lowest values for both bulk and tapped densities (Table 7). Based on the Carr index, all cements were classified as having very poor flowability, with the control cement displaying the highest Carr index value. In cements ground with GA1, GA2, GA3, and GA4, a reduction in the Carr index was observed as the additive dosage increased up to 0.05%; however, a further increase to 0.1% resulted in a higher Carr index. In contrast, for cements containing GA5, the Carr index remained largely unaffected by increasing the dosage to 0.1% (Table 7).

According to the Hausner ratio, the control cement was classified as extremely poor, while the remaining cements were categorized as very poor. Similar to the Carr index, the Hausner ratio indicated that the grinding aids improved flowability at an optimum dosage, beyond which flowability declined.

Previous studies have reported that cements produced with GAs exhibit lower Carr index and Hausner ratio values than those without GAs, thereby enhancing powder flowability [33,34]. This enhancement has been attributed to the neutralization of surface charges and the development of smoother particle morphologies in GA-treated cements. In agreement with the literature, cements containing TEA-TIPA-based additives demonstrated lower Carr index and Hausner ratio values compared to the control. While the finer particle size of GA-containing cements increases surface energy and promotes agglomeration [23], the adsorption of GA molecules onto cement particle surfaces enhances dispersion and mitigates agglomeration, thereby improving powder flowability [35]. These two competing effects occur simultaneously. Among the formulations tested, GA5 yielded the most favorable powder flowability performance, consistent with the interaction of these opposing mechanisms.

#### 3.3.2. Angle of Repose

**Figure 6:**
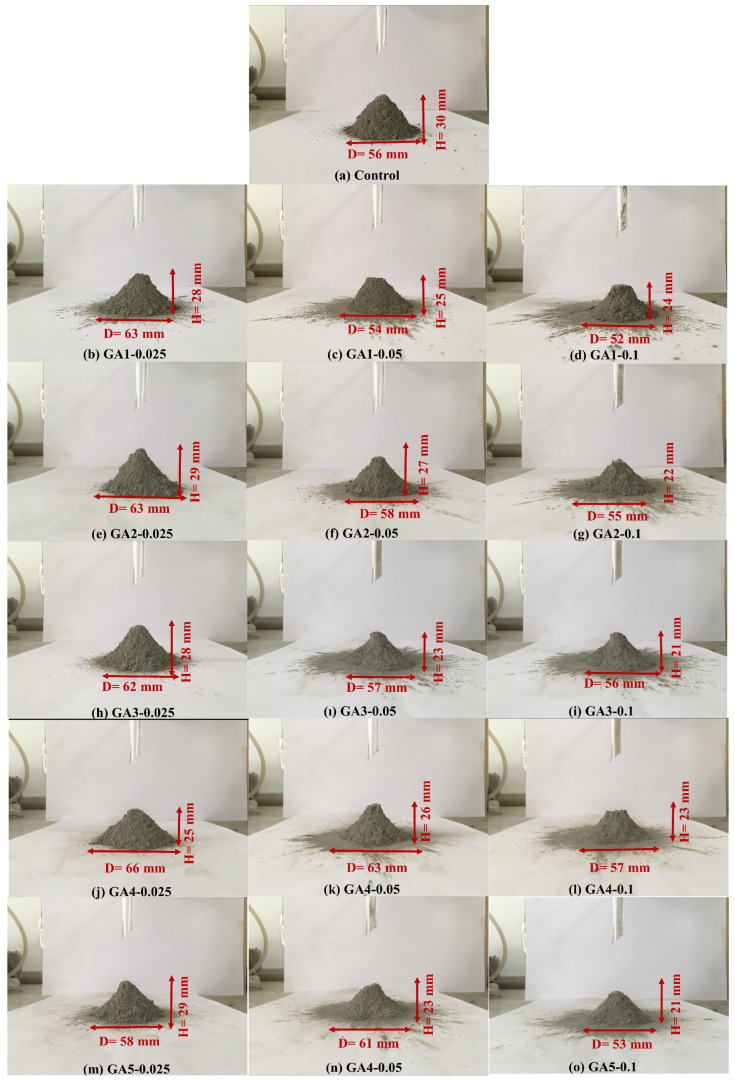
Angles of repose of control cement and cements containing GA at all dosages.

The angle of repose was measured to evaluate and compare the powder flowability of the produced cements. Table 8 presents the angles of repose for all cement samples, including both GA-free and GA-containing variants. Representative images of the control cement and the GA-containing cements after the angle of repose test are shown in Figure 6.

With the use of GAs, reductions in the angle of repose were observed relative to the control cement, ranging from a negligible 4% (GA5-0.025) to as much as 38% (GA4-0.1), indicating enhanced powder flowability. This improvement was attributed to several factors, including the grinding of sharp edges of larger particles to achieve a consistent Blaine fineness, the mitigation of particle agglomeration due to additive adsorption onto grain surfaces, and the formation of a narrower particle size distribution, as noted in the literature [36,37,38].

The reductions in the angle of repose obtained with GA1 (100% TEA) and GA2 (25% TEA, 75% TIPA) were not significantly influenced by dosage variation, with decreases of 9–11% and 6–9%, respectively, compared to the control (Table 8). However, as the TIPA content increased in the GA mixtures, further reductions in the angle of repose became more marginal. In the case of GA3, decreases of 10–22% were recorded, while GA4 led to more substantial reductions, ranging from 16% to 38% (Table 8). These findings confirm that the synergistic effects of TEA–TIPA mixtures, particularly those with higher TIPA content, contribute to improved powder flowability. This behavior has been attributed to TIPA’s ability to dissolve agglomerates [22] and TEA’s role in reducing particle agglomeration through enhanced electrostatic repulsion [29], as previously discussed. In contrast, the GA5 formulation (100% TIPA) yielded a relatively limited reduction in angle of repose, ranging from 4% to 21% compared to the control.

#### 3.3.3. Cement Morphologies

**Figure 7:**
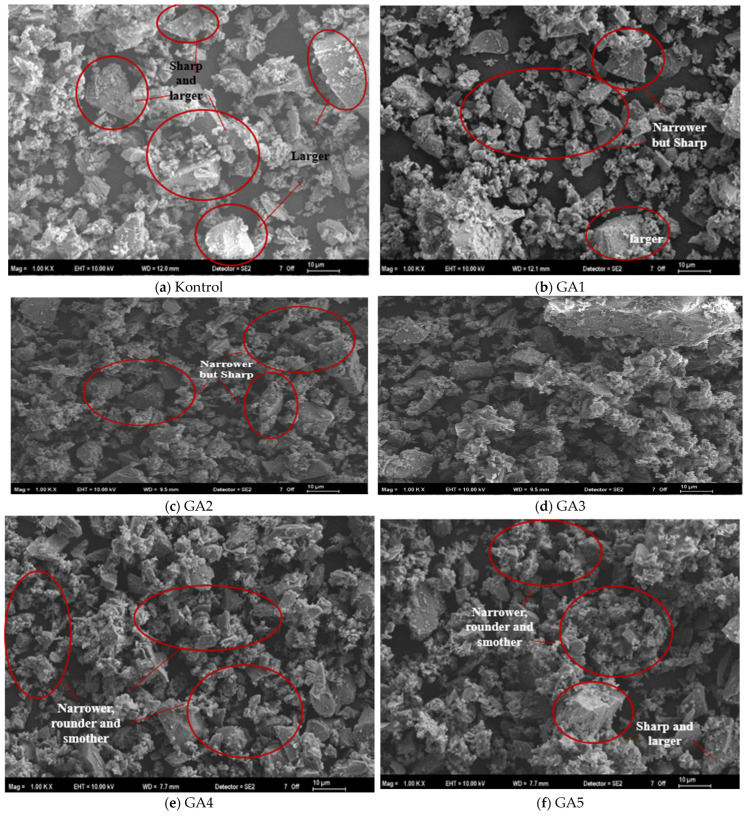
SEM images of control and cements containing 0.05% GA: (**a**) control, (**b**) GA1, (**c**) GA2, (**d**) GA3, (**e**) GA4, and (**f**) GA5.

SEM images of the control cement and the cements produced in the presence of GA at a dosage of 0.05% are given in Figure 7.

As illustrated in Figure 7a, the particles in the control cement appear larger, rougher, and more angular. In contrast, the cements incorporating grinding aids (GAs)—particularly GA3 and GA4, which contain higher proportions of TIPA—exhibit a narrower particle size distribution, along with smoother and more rounded grain morphologies. When Figure 7b,c are examined, it is evident that GA1 and GA2 cements possess relatively larger grains than GA4, and the smaller grains present more pronounced angular features and sharper edges. The SEM micrographs are in good agreement with the particle size distribution (Figure 5) and angle of repose (Figure 6) data. Furthermore, Figure 7e,f indicate that GA4 and GA5 cements exhibit a denser microstructure, consistent with their narrower grain size distributions. Nonetheless, a slightly higher presence of coarser and more angular particles can be observed in GA5 compared to GA4. Overall, the agglomeration in GA4 cement appears to have been effectively mitigated through the adsorption of the additive onto particle surfaces, which neutralized electrostatic charges. This led to the formation of finer, smoother, and more spherical particles, as confirmed by the SEM observations.

In conclusion, considering powder flowability and cement properties, GA4-prepared mixtures appear more suitable for efficient packaging and, owing to their narrower particle size distribution, may achieve higher early and long-term strength.

### 3.4. Taguchi Analysis Results

#### 3.4.1. Signal-to-Noise (S/N) Ratio Analysis

Grinding efficiency values were experimentally obtained for each combination of control factors defined by the experimental design. The collected data were then analyzed using the Taguchi method, and the grinding performance parameters were optimized by calculating the corresponding signal-to-noise (S/N) ratios. Given that grinding performance is a critical indicator of grinding efficiency and that higher values are desirable, the “larger-is-better” criterion was employed for the S/N ratio calculations. The calculated S/N ratios and corresponding mean values for strength parameters are presented in Table 9.

The effect of each control factor on grinding performance was analyzed using an “S/N response table.” The results of the S/N response analysis for grinding performance are presented in Table 10. 

**Figure 8:**
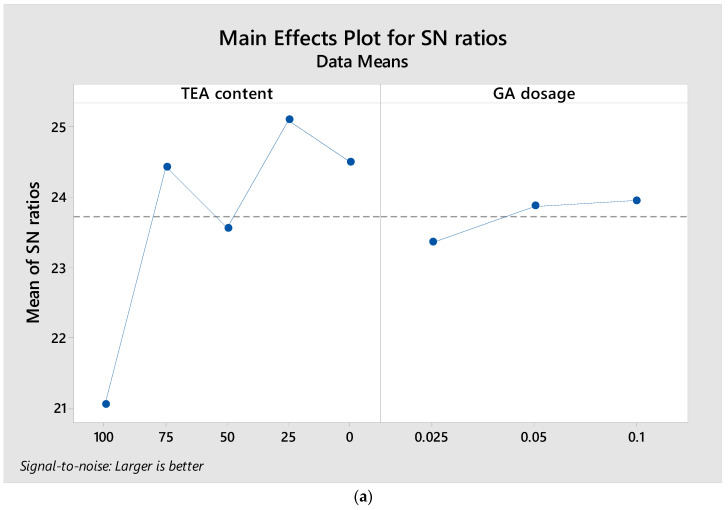

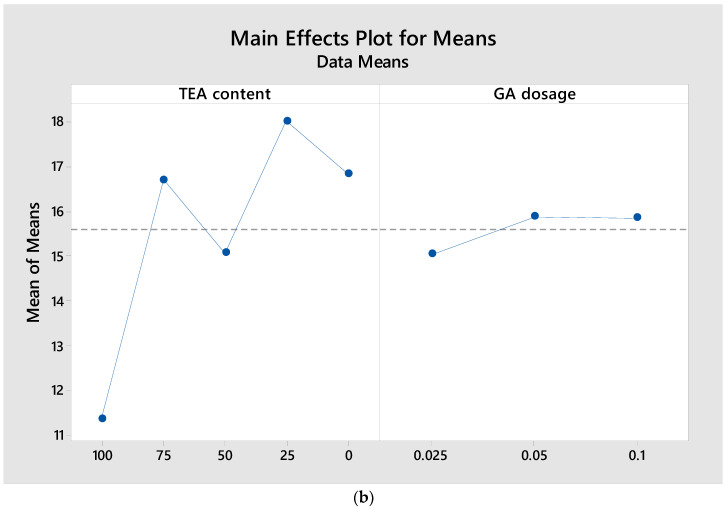
(**a**) Optimum parameters for S/N; (**b**) optimum parameters for means.

Table 10, prepared utilizing the Taguchi technique, presents the optimum levels of control factors for achieving enhanced grinding performance. The ideal processing parameters for the GAs were identified based on the highest signal-to-noise (S/N) ratios associated with each factor level. Accordingly, the optimal conditions for maximizing grinding efficiency were Level 4 for Factor A (75% TIPA, 25% TEA) and Level 3 (0.01%) for Factor B. These findings are further illustrated in Figure 8. The S/N response reveals a strong dependence on TEA content. Specifically, the S/N improves from 21.05 at 100% TEA to a maximum of 25.11 at 25% TEA (i.e., 75% TIPA), followed by a slight decline to 24.51 at 0% TEA (Table 10). The corresponding delta for TEA content is 4.06 (rank 1), whereas the dosage factor yields a much smaller delta of 0.59 (rank 2). These trends indicate that progressively reducing the TEA fraction and shifting toward a TIPA-dominant blend systematically increases the S/N ratio up to the 25% TEA/75% TIPA condition, beyond which further removal of TEA does not improve performance.

#### 3.4.2. ANOVA Method

In this study, ANOVA was applied to assess the impacts of GA type and dosage on grinding performance. The ANOVA results for grinding efficiency are summarized in Table 11. The analyses were conducted at a 5% significance level and 95% confidence interval. The influence of each control factor was evaluated by comparing its F-value, while a *p*-value below 0.05 indicated statistical significance. The final column of Table 11 presents the effect ratio, denoting the percentage contribution of each control parameter to the overall process performance.

According to Table 11, the TEA content and dosage in the grinding aid contributed 82.18% and 2.33%, respectively, to the grinding performance. Based on these results, the TEA content in the additive was identified as the most influential parameter affecting grinding performance. The ANOVA analysis calculated the model’s margin of error as 15.49%. TEA content is statistically significant for grinding efficiency (F = 10.61, *p* = 0.003) and explains ~82.18% of the variance, whereas dosage is not significant (F = 0.60, *p* = 0.571) with a contribution of ~2.33%. The remaining ~15.49% is attributed to experimental error/unexplained variation. These results substantiate that mixture ratio (expressed via TEA content in the TEA/TIPA blend) is the governing factor, while dosage changes within the tested range do not exert a statistically meaningful influence on the response.

#### 3.4.3. Limitation of Taguchi Analysis

The Taguchi design efficiently screens factors but primarily estimates main effects; potential interactions among factors are aliased and thus cannot be fully resolved without supplemental experiments. S/N-based optimization assumes stable variance and independent errors; deviations (e.g., heteroscedasticity, correlated measurements) may bias the ranking. Moreover, small sample sizes reduce power and increase sensitivity to experimental noise, elevating Type II error risk for weaker effects (e.g., dosage). To mitigate these limitations, confirmatory runs at the predicted optimum, randomization and replication, residual diagnostics, and distribution-free uncertainty quantification (e.g., bootstrap confidence intervals) are recommended; future work will incorporate these steps.

## 4. Conclusions

This study investigated the effects of TEA-TIPA mixtures on grinding efficiency, environmental impact, particle size distribution, adsorption behavior, powder flowability, and cement morphology. Parameters influencing grinding performance were analyzed using Taguchi and ANOVA methods. The key findings from the experiments and analyses are summarized below:

Mixing TEA and TIPA at specific ratios enhanced both grinding efficiency and environmental performance. In particular, the grinding aid composed of 75% TIPA and 25% TEA emerged as the most effective additive, delivering superior grinding efficiency, reduced CO_2_ emissions, and significant energy savings. Molecular modeling of Ca^2+^ ion adsorption by this additive supported the experimental observations, highlighting the synergistic effect achieved by combining the additives while mitigating their individual drawbacks.

Similarly, evaluations of powder flowability and cement morphology showed that TEA-TIPA mixtures improved performance. The 75% TIPA–25% TEA combination yielded a narrower grain size distribution, smoother and rounder particles, a reduced angle of repose, and enhanced powder flowability.

Taguchi and ANOVA analyses further confirmed that the combined use of grinding aids was the most influential factor affecting grinding performance.

Overall, the optimized use of TEA and TIPA at these ratios offers a promising approach for more sustainable and efficient cement production.

## Figures and Tables

**Table 1:** Physical and chemical properties of clinker and gypsum used in the study.

	Oxide Composition (%)	
	Clinker	Gypsum
SiO_2_	21.52	4.98
Al_2_O_3_	5.43	1.21
Fe_2_O_3_	3.31	0.83
CaO	65.38	28.94
MgO	1.04	0.83
SO_3_	0.38	39.67
Na_2_O + 0.658 K_2_O	0.83	0.37
Cl	0.01	-
Combined Water (T < 230°)	-	18.93
Other. %	1.58	4.24
C_3_S	56.51	
C_2_S	19.06	
C_3_A	8.79	
C_4_AF	10.07	
Loss of ignition	0.52	

**Table 2:** Factor parameters and their levels.

Parameters	Symbol	Levels 1	Levels 2	Levels 3	Levels 4	Levels 5
TEA content	A	100	75	50	25	0
GA Dosage	B	0.025	0.05	0.1		

**Table 3:** Optimized energies and interaction energies of TEA, TIPA, TEA-TIPA molecules, and molecules adsorbed with Ca^2+^ ion obtained by semiempirical/pm6 method.

Compound	Energy (a.u.)	ΔE_interaction_ (a.u.)
TEA-TEA	−0.461776	
TEA-TEA with Ca^2+^	−0.066429	0.395347
TEA-TIPA	−0.504772	
TEA-TIPA with Ca^2+^	−0.102427	0.402345
TIPA-TIPA	−0.551546	
TIPA-TIPA with Ca^2+^	−0.157568	0.393978

**Table 4:** Energy efficiencies of GAs used in different types and ratios.

	Blaine Fineness (cm^2^/g)	Number of Revolutions Required for the Target BLAINE	Grinding Time to Target Blaine (min)	Energy Consumed (kWh)	Relative Energy Efficiency (%)
Control	3800	7600	89.41	37.54	
GA1-0.025	3700	6860	80.71	33.88	9.74
GA1-0.05	3780	6740	79.29	33.29	11.32
GA1-0.1	3700	6610	77.76	32.65	13.03
GA2-0.025	3740	6340	74.59	31.31	16.58
GA2-0.05	3700	6220	73.18	30.72	18.16
GA2-0.1	3680	6430	75.65	31.76	15.39
GA3-0.025	3710	6450	75.88	31.86	15.13
GA3-0.05	3680	6520	76.71	32.20	14.21
GA3-0.1	3700	6390	75.18	31.56	15.92
GA4-0.025	3770	6210	73.06	30.67	18.29
GA4-0.05	3710	6150	72.35	30.38	19.08
GA4-0.1	3780	6330	74.47	31.27	16.71
GA5-0.025	3780	6420	75.53	31.71	15.53
GA5-0.05	3800	6330	74.47	31.27	16.71
GA5-0.1	3710	6210	73.06	30.67	18.29

**Table 5:** Energy efficiency, carbon footprint, and environmental impact achieved through the use of GAs.

Energy Efficiency and CO_2_ Emissions	Amount of Cement Produced Annually in Turkey, Kiloton	Energy Consumed in the Grinding Phase in the Production of 1 Ton of Cement, kWh	Total Energy Consumed in Clinker Grinding in Turkey, GWh	Provided Energy Savings, GWh	The Amount of CO_2_ Released from the Electricity Consumed During Grinding, in Kiloton	Amount of Avoided CO_2_ Emissions, Kiloton	Number of Trees that can Capture the Amount of CO_2_ Released (Million)	Number of Families Whose Annual Electricity Needs Can Be Met with the Saved Electricity (Thousand)
Kontrol	83,648	37.54	3140.15		1501.0		125.08	
GA1-0.025	83,648	33.88	2833.99	306.15	1354.6	146.3	112.89	61.23
GA1-0.05	83,648	33.29	2784.64	355.50	1331.1	169.9	110.92	71.10
GA1-0.1	83,648	32.65	2731.11	409.04	1305.5	195.5	108.79	81.81
GA2-0.025	83,648	31.31	2619.02	521.13	1251.9	249.1	104.32	104.23
GA2-0.05	83,648	30.72	2569.67	570.48	1228.3	272.7	102.36	114.10
GA2-0.1	83,648	31.76	2656.66	483.49	1269.9	231.1	105.82	96.70
GA3-0.025	83,648	31.86	2665.03	475.12	1273.9	227.1	106.16	95.02
GA3-0.05	83,648	32.2	2693.47	446.68	1287.5	213.5	107.29	89.34
GA3-0.1	83,648	31.56	2639.93	500.22	1261.9	239.1	105.16	100.04
GA4-0.025	83,648	30.67	2565.48	574.66	1226.3	274.7	102.19	114.93
GA4-0.05	83,648	30.38	2541.23	598.92	1214.7	286.3	101.23	119.78
GA4-0.1	83,648	31.27	2615.67	524.47	1250.3	250.7	104.19	104.89
GA5-0.025	83,648	31.71	2652.48	487.67	1267.9	233.1	105.66	97.53
GA5-0.05	83,648	31.27	2615.67	524.47	1250.3	250.7	104.19	104.89
GA5-0.1	83,648	30.67	2565.48	574.66	1226.3	274.7	102.19	114.93

**Table 6:** Powder flowability scale [32,33].

Flowability Classification	Carr Index	Hausner Rate	Repose Angle [°]
Excellent	≤10	1.0–1.11	25–30
Good	11–15	1.12–1.18	31–35
Fair	16–20	1.19–1.25	36–40
Passable	21–25	1.26–1.34	41–45
Poor	26–31	1.35–1.45	46–55
Very Poor	32–37	1.46–1.59	56–65
Extremely Poor	˃38	˃1.60	˃66

**Table 7:** Some powder fluidity values of cements.

	Bulk Density (kg/m^3^)	Apparent Density (kg/m^3^)	Carr Index	Hausner Rate
Control	781.3	1250.0	37.50	1.600
GA1-0.025	862.1	1315.8	34.48	1.526
GA1-0.05	892.9	1351.4	33.93	1.514
GA1-0.1	961.5	1470.6	34.62	1.529
GA2-0.025	806.5	1250.0	35.48	1.550
GA2-0.05	862.1	1282.1	32.76	1.487
GA2-0.1	925.9	1388.9	33.33	1.500
GA3-0.025	833.3	1315.8	36.67	1.579
GA3-0.05	892.9	1351.4	33.93	1.514
GA3-0.1	925.9	1428.6	35.19	1.543
GA4-0.025	806.5	1250.0	35.48	1.550
GA4-0.05	862.1	1282.1	32.76	1.487
GA4-0.1	892.9	1351.4	33.93	1.514
GA5-0.025	819.7	1219.5	32.79	1.488
GA5-0.05	877.2	1315.8	33.33	1.500
GA5-0.1	925.9	1388.9	33.33	1.500

**Table 8:** Angles of repose of the produced cements.

	Dosages (%bwoc)	Control	GA1	GA2	GA3	GA4	GA5
Angle of repose (°)	0.025	46.97	41.63	42.63	42.09	37.15	45.00
	0.05		42.80	42.95	38.90	39.54	37.02
	0.1		42.71	44.36	36.87	28.90	38.40

**Table 9:** Test results, S/N ratios, and mean values.

Test No.	Control Factors		Grinding Efficiency (%)	S/N Ratio for Grinding Efficiency	Means for Grinding Efficiency
	TEA Content	GA Dosage			
1	100	0.025	9.74	19.7712	9.74
2	100	0.05	11.32	21.0769	11.32
3	100	0.1	13.03	22.2989	13.03
4	75	0.025	16.58	24.3917	16.58
5	75	0.05	18.16	25.1823	18.16
6	75	0.1	15.39	23.7448	15.39
7	50	0.025	15.13	23.5968	15.13
8	50	0.05	14.21	23.0519	14.21
9	50	0.1	15.92	24.0389	15.92
10	25	0.025	18.29	25.2443	18.29
11	25	0.05	19.08	25.6116	19.08
12	25	0.1	16.71	24.4595	16.71
13	0	0.025	15.53	23.8234	15.53
14	0	0.05	16.71	24.4595	16.71
15	0	0.1	18.29	25.2443	18.29

**Table 10:** S/N response results of grinding performance.

Response Table for Signal-to-Noise Ratios			Response Table for Means		
Level	TEA Content	GA Dosage	Level	TEA Content	GA Dosage
1	21.05	23.37	1	11.36	15.05
2	24.44	23.88	2	16.71	15.9
3	23.56	23.96	3	15.09	15.87
4	25.11		4	18.03	
5	24.51		5	16.84	
Delta	4.06	0.59	Delta	6.66	0.84
Rank	1	2	Rank	1	2

**Table 11:** ANOVA results for grinding performance.

Source	Degree of Freedom (DoF)	Sum of Squares (SS)	Mean Square (MS)	F-Value	*p*-Value	Effect Ratios (%)
TEA content	4	80.638	20.16	10.61	0.003	82.18
GA dosage	2	2.287	1.144	0.6	0.571	2.33
Error	8	15.202	1.9			15.49
Total	14	98.128				100.00

## Data Availability

The original contributions presented in this study are included in the article. Further inquiries can be directed to the corresponding author.
